# In Vitro Characterization of Hydroxyapatite-Based Coatings Doped with Mg or Zn Electrochemically Deposited on Nanostructured Titanium

**DOI:** 10.3390/biomimetics9040244

**Published:** 2024-04-18

**Authors:** Diana M. Vranceanu, Elena Ungureanu, Ionut C. Ionescu, Anca C. Parau, Vasile Pruna, Irina Titorencu, Mihaela Badea, Cristina-Ștefania Gălbău, Mihaela Idomir, Mihaela Dinu, Alina Vladescu (Dragomir), Cosmin M. Cotrut

**Affiliations:** 1 Faculty of Materials Science and Engineering, National University of Science and Technology Politehnica Bucharest, 313 Splaiul Independentei, 060042 Bucharest, Romania; diana.vranceanu@upb.ro (D.M.V.);; 2 National Institute of Research and Development for Optoelectronics INOE2000, 409 Atomistilor, 077125 Magurele, Romania; 3 Romanian Academy Institute of Cellular Biology and Pathology “Nicolae Simionescu”, 8 B.P. Hasdeu, 050568 Bucharest, Romania; 4 Prophylactic and Clinical Disciplines, Department of Fundamental, Faculty of Medicine, Transilvania University of Brasov, 56 Nicolae Balcescu, 500019 Brasov, Romania; 5 Research Center for Fundamental Research and Prevention Strategies in Medicine, Research and Development Institute, Transilvania University of Brasov, Romania Institutului, 10, 500484 Brașov, Romania

**Keywords:** coatings, biofunctionalization, doped hydroxyapatite, electrochemical behavior, biocompatibility, antibacterial

## Abstract

Biomaterials are an important and integrated part of modern medicine, and their development and improvement are essential. The fundamental requirement of a biomaterial is found to be in its interaction with the surrounding environment, with which it must coexist. The aim of this study was to assess the biological characteristics of hydroxyapatite (HAp)-based coatings doped with Mg and Zn ions obtained by the pulsed galvanostatic electrochemical method on the surface of pure titanium (cp-Ti) functionalized with titanium dioxide nanotubes (NTs TiO_2_) obtained by anodic oxidation. The obtained results highlighted that the addition of Zn or Mg into the HAp structure enhances the *in vitro* response of the cp-Ti surface functionalized with NT TiO_2_. The contact angle and surface free energy showed that all the developed surfaces have a hydrophilic character in comparison with the cp-Ti surface. The HAp-based coatings doped with Zn registered superior values than the ones with Mg, in terms of biomineralization, electrochemical behavior, and cell interaction. Overall, it can be said that the addition of Mg or Zn can enhance the *in vitro* behavior of the HAp-based coatings in accordance with clinical requirements. Antibacterial tests showed that the proposed HAp-Mg coatings had no efficiency against Escherichia coli, while the HAp-Zn coatings registered the highest antibacterial efficiency.

## 1. Introduction

A worldwide issue that the healthcare system is confronting nowadays is due to implant-associated infections, which have increased since the number of surgical interventions aiming to replace different body parts with implants has also increased [1,2]. Moreover, considering the rising age of the general population, it is estimated that the number of implant-associated infection revisions will increase because of growing concern over antibiotic-resistant bacteria [3,4].

In most cases, biofilm appearance induces an inflammatory response or bone damage, and, therefore, to prevent biological complications, it is necessary to decrease early bacterial attachment [5]. The driving factor behind bacteria adhesion or inhibition consists of the surface features of the implantable material [6,7]. Thus, surface properties are accountable for the fate of an implant since they are strongly connected to the osseointegration process. In this regard, the initial stability of an implant is achieved at the surgical site and is followed by secondary stability, which is reached during the healing process [8]. Therefore, the osseointegration process can be viewed as a race between the microorganism that seeks to colonize and form the biofilm versus the endogenous tissue that seeks to grow on the implant surface, indicating that the most common complications associated with implants begin at the hard tissue–implant interface [7].

In medical applications, titanium and its alloys continue to prevail since these biomaterials exhibit great mechanical properties, excellent corrosion resistance, and high biocompatibility [5,6,9]. However, their poor osseointegration ability and lack of antibacterial effect cannot be neglected [10,11] since, at the global level, more and more surface modifications are being used to overcome these disadvantages [12,13].

Thus, one of the obstacles to successful implantation can be addressed by using surface engineering techniques to design a surface that can satisfy both antibacterial and biocompatibility requirements [14,15]. Among the proposed surface biofunctionalization solutions are subtractive methods [16,17], such as acid etching, sandblasting, large-grit sandblasting, and acid etching, also known as SLA, laser methods, and anodization and also additive methods, of which the most used are plasma treatment [18] and the deposition of coatings obtained by a large variety of methods, namely physical, chemical, and electrochemical [19,20]. Of all the proposed methods, it appears that the coatings field is the one through which a wider variety of properties and clinical requirements can be fulfilled.

One of the most used coating materials is hydroxyapatite (HAp) because of its similarity to natural apatite found in human hard tissue, as the main component of the inorganic phase [20,21,22,23]. Moreover, because of its structure, HAp allows the addition of several other biocompatible elements such as Mg, Sr, Zn, and Ag, enabling other properties and characteristics of the coatings [20,24,25,26]. Thus, doping elements, such as Ag, Zn, and Cu, are known to have a bactericidal effect [26,27,28,29,30], while Sr is known to support bone growth in osteoporotic bone [31,32], and Mg can enhance the biodegradability of HAp-based coatings [33,34,35,36].

Of course, special attention must be given to the amounts of each doping element since a quantity other than optimum can negatively impact the overall properties [37]. It has been established that Ag^+^ in a quantity of up to 6 at.% favors cell viability and proliferation [31,38], while at higher concentrations, it has been reported to be cytotoxic [39,40]. Another element with a bactericidal effect is Zn^2+^, which, in percentages between 0.4 and 6% wt. [24,41,42], is efficient against *K. pneumoniae*, *S. aureus*, *B. cereus*, and *E. coli* strains without inducing cytotoxicity. Regarding Mg presence in the HAp structure, the literature has pointed out its positive impact on osseointegration ability and an enhancement in the coating’s solubility and biodegradation [37,43]. Unless it is substituted with other ions such as fluoride and carbonate, the incorporation of Mg^2+^ within the HAp structure is limited to 0.4–2% [44]. Furthermore, some studies have shown that when the amount of Mg^2+^ from the implanted material exceeds the one found in bacterial cells (15–30 mM), Mg displays an antibacterial effect [45].

HAp-based coatings undoped or doped can be deposited on metallic materials, namely, Ti and its alloys, by physical methods such as plasma spraying (PS) [46], magnetron sputtering (MS) [47], and pulsed laser deposition (PLD) [48], and chemical methods such as the sol-gel route [49], or electrochemical methods, namely, electrophoretic [50] or electrochemical deposition [51]. The electrochemical deposition method is a versatile technique, which, in comparison with other deposition methods, can be used to coat complex 3D shapes, the electrochemical parameters involved in the deposition process can be easily varied, and coatings can be obtained at low temperatures (below 90 °C) and from a wide variety of electrolytes [51,52,53]. Nonetheless, the poor adhesion of electrochemically deposited HAp coatings can be increased with the addition of doping elements [54] and/or by modifying the substrate with titanium dioxide nanotubes (NTs TiO_2_), which can offer anchorage for the coating [55,56].

The current study is a follow-up of our previous research [57,58] in which HAp-based coatings doped with two different concentrations of Mg and Zn electrochemically deposited on nanostructured titanium dioxide nanotubes surface were investigated in terms of physico-chemical properties. Table 1 summarizes the main physico-chemical properties of the developed HAp-based coatings electrochemically deposited on the cp-Ti substrate functionalized with titanium dioxide nanotubes (NTs TiO_2_) [57,58]. Thus, criteria such as the Ca/P ratio, the amount of the doping element found in the HAp coating, coating thickness, and coating adhesion were used to select the coatings for further testing *in vitro*.

Based on the results previously obtained (Table 1), the samples codified as H-Mg2 and H-Zn2, which had higher amounts of Mg and Zn, were excluded from the current study because of their differences in terms of thickness vs. the undoped HAp and their poorer adhesion to the substrate. On the other hand, the coatings codified as H-Mg1 and H-Zn1 had comparable thicknesses and adhesion to the substrate, which made them suitable for further testing. 

In comparison with other studies available in the literature on the same topic, the current research aims to offer a detailed overview of the *in vitro* behavior of hydroxyapatite-based coatings undoped and doped with Mg or Zn electrochemically deposited on a pure titanium surface functionalized with titanium dioxide nanotubes by anodic oxidation in terms of wettability, electrochemical behavior, and bioactivity in specific acellular media, coupled with biocompatibility assays and antibacterial efficiency. Furthermore, the influence of each doping element (Mg and Zn) is also taken into consideration in order to offer a new perspective on this type of coating and its potential in medical applications.

## 2. Materials and Methods

### 2.1. Sample Preparation and Initial Results

**Figure 1:**
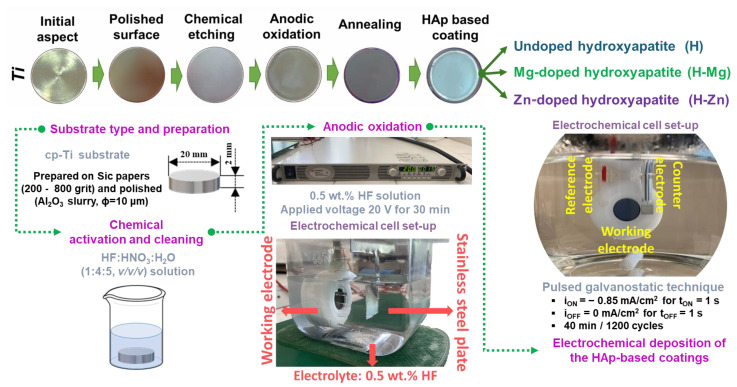
Schematic illustration of the Ti biofunctionalization stages.

Details regarding the sample preparation and the electrochemical deposition of the coatings, namely, the preparation of the cp-Ti substrate nanostructured with titanium dioxide nanotubes (NTs) uncoated and coated with osteoconductive HAp-based coatings are available in Refs. [57,58]. A succinct overview of the biofunctionalization phases and the key variables are shown in Figure 1. Figure 1 also shows the macroscopic aspect of the samples following each step of preparation. 

Briefly, a cp-Ti bar purchased from Bibus Metals AG, Germany, with a diameter of 20 mm was used to cut discs with a height of 2 mm. The resulting discs were prepared on metallographic papers of different grits (200 ÷ 800) and degreased with acetone in an ultrasonic bath (Bandelin, Berlin, Germany) for approx. 30 min. The anodic oxidation method was applied to obtain the nanostructured titanium dioxide nanotubes (NTs TiO_2_) by using a two-electrode electrochemical cell set-up and a 0.5 wt.% HF electrolyte (Figure 1). The voltage was set to 20 V and was maintained constant with a DC power supply system (N5771A model, Keysight, Böblingen, Germany) for 30 min. Then, the samples were annealed in a heat treatment furnace (N17/HR model, Nabertherm, Lilienthal, Germany) at 450 °C for 120 min. 

The samples obtained at this stage were used as substrates for the coating’s deposition. Thus, the electrochemical deposition of the hydroxyapatite undoped and doped with Mg or Zn was carried out in a three-electrode electrochemical cell (Figure 1), and the process was controlled with a multichannel Potentiostat/Galvanostat (Parstat MC, Princeton Applied Research–Ametek, Oak Ridge, TN, USA) by applying the pulsed galvanostatic technique. 

Figure 1 presents the main electrochemical parameters used, while Table 2 presents the chemical composition of the electrolyte along with the sample codification. The deposition process was conducted at a temperature of 75 °C, which was maintained constant with a basic magnetic hotplate stirrer (KA RCT Basic Safety Control Hotplate/Stirrer and ETS-D6 Temp, IKA, Staufen, Germany) which also helped to continuously stir the electrolyte. 

### 2.2. Characterization and Testing

#### 2.2.1. Morphology and Phase Composition

Using a scanning electron microscope (SEM), Phenom ProX (Phenom World, Eindhoven, The Netherlands), the morphology of the developed materials was examined both before and after *in vitro* tests.

X-ray diffraction patterns (XRD) were used to evaluate the phase composition of the coatings, using a SmartLab diffractometer (Rigaku), equipped with a 9 kW Cu rotating anti-cathode, a vertical goniometer of 300 mm radius with sample horizontal mount, and a HyPix 3000 two-dimensional semiconductor detector. The 2θ/θ diffractograms were recorded over a 20°–80° range with a resolution of 0.03°. The diffractograms were analyzed using Rigaku’s PDXL software package (version 2).

#### 2.2.2. Surface Energy and Wettability

Using the sessile drop technique at 25 ± 0.5 °C and a KSV-Instruments Attention TL101 (Biolin Scientific, Gothenburg, Sweden), the contact angles of the samples were ascertained. A charge-coupled device (CCD) camera recorded the contour of each sample after the droplet containing 5 μL was applied with a typical Hamilton micro-syringe. To obtain reliable contact angle data, three droplets were deposited on different regions of each sample. Three distinct liquids with known surface tension components ($\gamma_{s}^{\;}$) were used to determine the surface free energy ($\gamma_{s}^{tot}$) of the developed materials. These liquids are listed in Table 3. Considering the surface tension parameters values given in the literature [60] for highly polar liquids (DW—distilled water) and for dispersive ones (EG—ethylene glycol and DI—diiodomethane), using the Fowkes approach [61,62], the total surface free energy $\gamma_{s}^{tot}$ of the samples was determined. 

When two phases are in equilibrium, the work of adhesion (W_adh_) is the reversible thermodynamic work required to separate the interface to an infinite separation distance. In a system of solid and liquid, the work of adhesion may be calculated using Young-Dupre’s equation (Equation (1)), which can be determined by measuring the liquid’s surface tension and contact angle as follows [63,64]:

(1) $$W_{adh}=\gamma_{SL}\left(1+cos\theta \right)$$

where the superscripts *d* and *p* denote the dispersive and polar components, $\gamma_{SL}^{\;}\;$ is the interfacial surface tension, $\gamma_{L}^{\;}\;$ is the surface energy (tension) of the liquid phase, and $\gamma_{S}^{\;}\;$ is the surface energy of the solid phase. 

#### 2.2.3. Electrochemical Behavior in Acellular Media

The coatings’ electrochemical activity was evaluated at 37 °C (±0.5 °C) in two artificial environments that resemble the human body as follows: (1) Fusayama artificial saliva (F-AS), which is comparable to natural saliva, and (2) simulated body fluid (SBF), which mimics human plasma. The two types of artificial environments were selected to analyze if the proposed materials are suitable for implants used in dentistry and also in orthopedics. A potentiostat/galvanostat PARSTAT 4000 (Princeton Applied Research, USA) equipped with a low current interface unit (LCI, Princeton Applied Research) was used to control the electrochemical tests. The electrochemical tests were conducted under a Faraday cage to eliminate the possible interferences induced by the electromagnetic field. A three-electrode cell set-up configured as following was used for these experiments. The working electrodes (WEs) were the investigated specimens, the reference electrode (RE) was a calomel electrode, and the counter electrode (CE) was a platinum foil. The chemical compositions of the electrolytes used to evaluate the electrochemical behavior are shown in Table 4.

#### 2.2.4. Bioactivity Tests

The proposed materials were tested for their ability to degrade in phosphate buffer solution (PBS) and for their ability to form new apatite precipitates in SBF through *in vitro* bioactivity tests. The samples were placed in an incubator (Memmert IF 55, Memmert GmbH, Schwabach, Germany) at human body temperature (37 ± 0.5 °C) for 21 days while submerged in the testing medium (SBF or PBS). Every day, the testing medium was changed to avoid chemical exhaustion and to ensure that no bacteria or other microorganisms could grow. An analytical balance with a 0.01 mg accuracy was used to monitor the mass evolution. The following equation was used to calculate the weight variation (Equation (2)):

(2) $$∆m=m_{f}-m_{i}$$

where the apatite mass formed on the surface in mg is represented by Δm, while the samples mass before and after exposure to SBF or PBS are represented by m_i_ and m_f_, respectively. The samples were removed from the testing media, rinsed in distilled water, and then dried for 24 h in a desiccator to eliminate any remaining water from the samples’ structures. For each period and testing medium, different samples were used. Following this procedure, the samples were weighed. Mass evolution was used to quantify the *in vitro* bioactivity results for both the coated and uncoated samples. Additionally, X-ray diffraction (XRD, Rigaku Smart Lab, Tokyo, Japan) and scanning electron microscopy (Phenom ProX, Phenom World, Eindhoven, The Netherlands) were used to highlight the phasic composition and surface morphology of the coatings following their immersion in the testing media. 

#### 2.2.5. *In Vitro* Biological Assay

##### Cell Seeding and Cell Morphology

Human osteosarcoma cell line MG63 (American Type Culture Collection) was used for the biological tests. After being sterilized for 24 h in 70% (*v*/*v*) ethanol, the samples were cleaned with sterile distilled water and then incubated for another 24 h in the culture medium. The cells were then cultured for three days in 1‰ glucose DMEM medium supplemented with 10% *v*/*v* fetal bovine serum and 1% of penicillin with streptomycin and neomycin. The culture conditions were as follows: the temperature was set to 37 °C, the relative humidity was 95%, and the CO_2_ concentration was 5% (*v*/*v*). The density of the seeded cells was 9000/cm^2^. Eight duplicates of each type of coating were used for the *in vitro* tests, and the assay was run twice.

The cells were fixed with 0.1% Triton X-100 (Sigma-Aldrich, St. Louis, MO, USA) and 4% paraformaldehyde for morphology evaluation. Following the guidelines provided by the manufacturer (Thermo Fisher Scientific, Waltham, MA, USA), the β actin filaments were stained with Phalloidin-FITC, and the nucleus was identified using DAPI (Sigma-Aldrich, St. Louis, MO, USA) and observed with a Zeiss Observer D1 microscope.

##### MTT Assay

The 3-(4,5-dimethylthiazol)-2,5-diphenyltetrazolium bromide (MTT) assay was used to measure the *in vitro* cellular viability of the samples to determine their biocompatibility after one and three days. The MTT test was used to assess cell viability, while actin staining was used to assess cell morphology. The MTT assay was performed for *in vitro* evaluation of cell metabolism level. After seeding the cells on the samples, they were rinsed with warm phosphate-buffered saline (PBS) solution and incubated for 3 h with 0.5 mg/mL MTT solution under cell culture conditions. The cellular mitochondrial dehydrogenase enzymes in metabolically active cells reduce the tetrazolium salt, producing an insoluble purple formazan that is subsequently soluble in 0.1N HCl in anhydrous isopropanol. The concentration of formazan solution, which is directly related to the cellular enzymatic reduction activity, was evaluated by determining the OD_570_ (optical density at λ = 570 nm) to 630 nm. A Tecan spectrophotometer was used to measure the absorbance at 570 nm, which is correlated with the quantity of viable and metabolically active cells.

#### 2.2.6. Antibacterial Efficiency 

*Escherichia coli* strains (ATCC 25922) were used to evaluate the antibacterial efficacy of the surfaces that were developed. A densitometer was used to prepare a stock suspension of *Escherichia coli* of 0.5 McFarland equivalent to 1.5 × 10^8^ CFU/mL. The stock solution was diluted to a concentration of 10^4^ CFU/mL. Following a 30-minute incubation period at room temperature, the testing samples were rinsed in 3 mL of NaCl 0.9% after being inoculated with the bacterial suspension (V = 113 μL). A volume of 100 μL was taken from the washing liquid and inoculated onto blood agar. 

Following a 24 h incubation period of the Petri dishes in the thermostat (37 °C), an automatic analyzer (InterScience Scan 300-Soft Scan InterScience, Saint Nom la Brétèche, France) was used to quantify the number of *Escherichia coli* colonies. The examinations were conducted in duplicate. The results were analyzed, and the average along with their standard deviations were computed. The antibacterial efficiency of the tested samples was calculated using the following equation (Equation (3)) [65,66]:

(3) $$Antibacterial\;efficiency=\frac{N_{C}-N_{S}}{N_{C}}\cdot 100(\%)$$

where N_C_ represents the number of colonies on the control samples and N_S_ represents the number of colonies on the tested samples.

## 3. Results and Discussion

### 3.1. Wettability and Surface Free Energy

Surface free energy (SFE) may be seen as a physical feature of a material that influences wettability and, as a result, its adhesion to the surrounding tissue. Surfaces with higher energies tend to improve the interaction between osteoblasts and the implanted material. Surface energy controls two important phenomena for biomaterial–cell interaction, namely, protein adsorption and cell adhesion. Implantable materials are initially covered by a layer of proteins, which is critical in mediating the cellular response [67].

**Figure 2:**
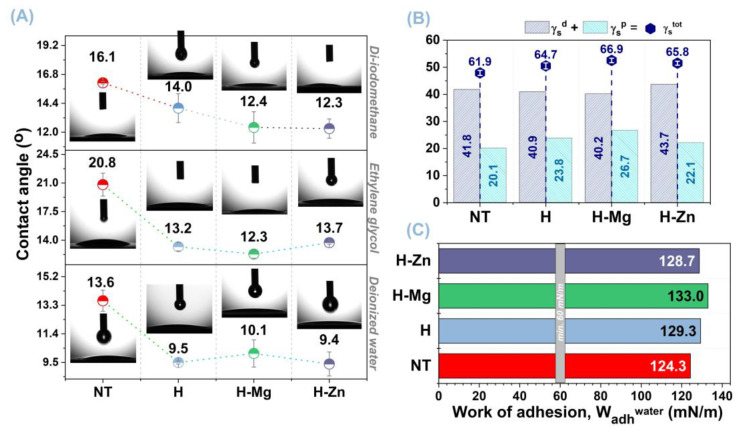
Evolution of the contact angle (**A**), surface free energy (**B**), and work of adhesion (**C**) for the examined materials.

Surface tension and energy were used to investigate wettability characteristics. The contact angle (CA) measurements of the three liquids employed are shown in Figure 2A, and the surface free energy values obtained for the examined materials are shown in Figure 2B. 

In the literature [9,68], Ti polished surfaces have been shown to offer contact angle values of 53.9° (±5.1) for distilled water, 52.2° for ethylene glycol, and 35.4° (±3.5) for diiodomethane. Based on this, it can be observed that the biofunctionalization of Ti by electrochemical processes, such as anodic oxidation and electrochemical deposition, has improved surface wettability since lower contact angle values were achieved for each liquid (Figure 2A). Therefore, regardless of the testing liquid, the uncoated NT TiO_2_ nanostructured surface reported CA values between 13.6° and 20.8°. A greater contact surface area results from an increase in the surface-to-volume ratio, which is associated with the higher CA values obtained in the case of the NT TiO_2_ nanostructured surface [69,70]. 

Significantly lower contact angle values by approx. 30% were obtained upon biofunctionalization of the NT nanostructured surface with undoped and Mg- or Zn-doped HAp-based coatings, suggesting that the developed coatings had a favorable effect on the NT surface’s wetting degree. When the CA values of the coatings doped with Mg or Zn are compared to those of the undoped HAp-based coatings, it is observed that all the values are within the same range, with no remarkable differences. Even though no significant differences were observed between the coatings doped with Mg and Zn, it was noted that in comparison with Mg, the addition of Zn resulted in a reduction in the contact angle for deionized water and di-iodomethane liquids and an increment in ethylene glycol. Nonetheless, in certain circumstances, the variations in the contact angle values for the coatings doped with magnesium or zinc can be disregarded, since they are within the error range indicated by the standard deviation computation. 

It can be concluded that the generated surfaces had a strong hydrophilic character, indicating a good degree of wetting since all CA values were smaller than 21°.

As previously stated, the values found for the surface free energy of the materials under investigation are shown in Figure 2B. A solid material’s surface free energy enables the measurement/quantification of intermolecular interactions at the interface, which influences the wetting degree, cell adsorption, and adhesion. Since the SFE generates strong attractive forces that will drag down the liquid droplets and improve the spreadability, such biomaterials with high surface free energy are also characterized by high wettability [71].

As reported in the literature [9,68], the polished Ti surface exhibits an SFE of 33.5 ÷ 49 mN/m when compared with the experimental materials examined in this study. This suggests that the proposed biofunctionalization techniques result in a notable increase in SFE. Also, the surface energy of Ti is improved by biofunctionalization with titanium dioxide nanotubes coated with HAp, from 61.9 mN/m for the NT surface to 64.7 mN/m for the undoped HAp (H) coatings.

It is observed that the addition of the doping elements to the HAp structure resulted in a slight improvement of the total surface free energy, reaching values of 65.8 mN/m for H-Zn and 66.9 mN/m for H-Mg. The H-Mg sample had the highest surface energy compared with the NT surface, followed by H-Zn and H, indicating that the addition of Mg or Zn improves the surface characteristics of the HAp-based coatings.

The changes in the SFE, consisting of its two components, dispersive and polar, of the investigated materials are a direct cause of the chemical composition, topography, morphology surface roughness, and/or surface chemistry of the investigated materials. In comparison with cp-Ti, which has a smooth surface, the morphology of the NT uncoated and coated with undoped and Mg- or Zn-doped HAp led to a higher roughness (Table 1), increasing the wetting area. In terms of surface chemistry, one major role was played by the presence of the hydroxyl and phosphate functional groups identified in the case of the HAp-based coatings, as demonstrated by Fourier-transform infrared spectroscopy [57,58], and the presence of titanium dioxide as anatase phase [57,58], in the case of the NT surface, enabling the surface wettability. 

According to the literature, the polar component of surface free energy governs cell proliferation and adhesion on the surface of implantable materials [72]. Bren et al. [73] investigated the effect of surface energy on the growth and adhesion of osteoblast cells on stainless steel and Ti6Al4V alloy and concluded that higher surface energy materials improve osteoblast differentiation. In the study performed by Grubova et al. [74], it was shown that HAp-based coatings had higher values of the polar component (γ_s_^p^) than that of the Ti substrate. Thereby, by examining the polar component (γ_s_^p^), it is possible to see that the HAp-based coatings have higher values (H, γ_s_^p^_=_ 23.8 mN/m) than the NT surface (NT, γ_s_^p^ = 20.1 mN/m). In addition, it is noted that the values of the polar component of the free energy for the H-Mg and H-Zn coatings exhibit values of 26.7 mN/m and 22.1 mN/m, respectively. This indicates that Mg leads to a greater value than Zn. All HAp-based coatings were therefore seen to increase the SFE polar component and are predicted to improve cell interactions.

Figure 2C presents the work of adhesion (W_adh_) values for water obtained for the investigated surfaces. It is known that a W_adh_ higher than 60 mN/m [75] favors adequate tissue adhesion to the surface of an implantable device. Moreover, the SFE of sanguine plasma is 52 mN/m [76], while the one for saliva can vary between 53.4 mN/m and 63.2 mN/m [77,78]. As a result, it is evident that the investigated surfaces—NT uncoated and coated with HAp undoped and doped with Mg or Zn—have values higher than 60 mN/m, indicating that biological fluids from the environment adjacent to implantable medical devices may easily wet them.

The results obtained also show good agreement with a study conducted by Nakamura et al. [79], wherein it was demonstrated that the cellular interaction is enhanced by HAp-based coatings with an SFE of approx. 130 mN/m.

### 3.2. Electrochemical Behavior in Synthetic Media

**Figure 3:**
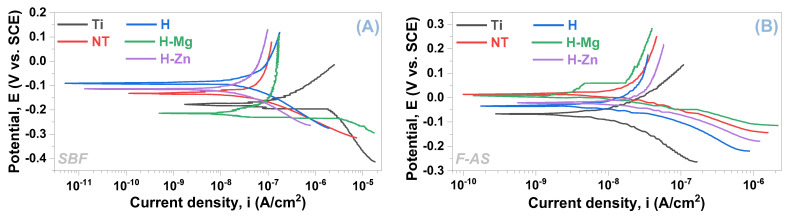
Tafel plots of the investigated materials in SBF (**A**) and F-AS (**B**).

The open circuit potential (E_OC_) was measured for 3 h during the linear polarization test, and the potentiodynamic slopes were registered at a scanning rate of 0.167 mV/s within a range of ±200 mV vs. E_OC_. The Tafel plots are shown in Figure 3, and Table 5 presents the main electrochemical parameters for both testing media that were determined using the Tafel extrapolation method. 

**Figure 4:**
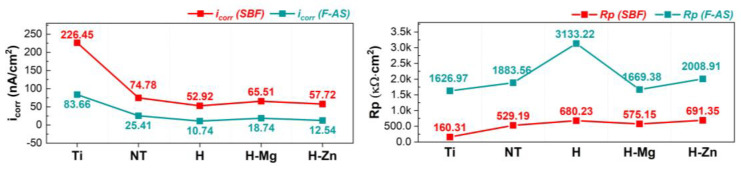
Evolution of the main electrochemical parameters (i_corr_—corrosion current density, Rp—polarization resistance) in SBF and F-AS.

A given material is resistant to corrosion when the corrosion potential (E_corr_) has a higher electropositive value, the polarization resistance (Rp) is high, and the corrosion current density (i_corr_) is small [80,81]. Based on these criteria, it is evident that the TiO_2_ nanostructured surface improved the electrochemical behavior of the Ti surface when compared with the unmodified Ti samples (Table 5). This is indicated by the higher Rp and the lower i_corr_ that were registered in both SBF and F-AS media (Table 5 and Figure 4).

Table 5 highlights that the HAp-based coatings improved the electrochemical behavior of the nanostructured surfaces by exhibiting lower values of the corrosion current densities (i_corr_) in comparison with the nanostructured surface (NT), indicating that the coatings have a protective character, irrespective of the electrolyte’s type. Notably, all HAp-based coatings exhibit higher values of the polarization resistance (Rp) parameter.

The evolution of the i_corr_ and Rp parameters for the materials under investigation in the two artificial physiological environments is shown in Figure 4. 

Regarding how the doping element affects electrochemical behavior, the following can be stated. The addition of Mg results in a lower polarization resistance and an increase in corrosion current density when compared with undoped HAp (H) in both testing environments (SBF and F-AS), suggesting that these coatings have a poorer electrochemical behavior. On the other hand, the addition of Zn into the HAp-based coatings results in a higher corrosion current density than the undoped HAp but smaller than the value of the H-Mg coatings, irrespective of the testing medium (SBF or F-AS). According to an analysis of the polarization resistance in both environments, it is noted that the H-Zn coatings exhibit higher values than those of H-Mg, regardless of the testing medium, but lower values than HAp in F-AS and higher than HAp in SBF.

As a result, the addition of Mg results in poorer electrochemical behavior of HAp, which is due to the nature of this element, namely, its high reactivity in aqueous media. According to these findings, it can be assumed that the degradation capacity of HAp can be controlled by adding Mg. In contrast, the addition of Zn to the HAp structure improves the electrochemical behavior when compared with Mg-doped HAp coatings, because Zn degrades at a lower rate than Mg. To summarize, the electrochemical properties of HAp-based coatings can be modulated by incorporating Mg or Zn into the HAp structure.

**Figure 5:**
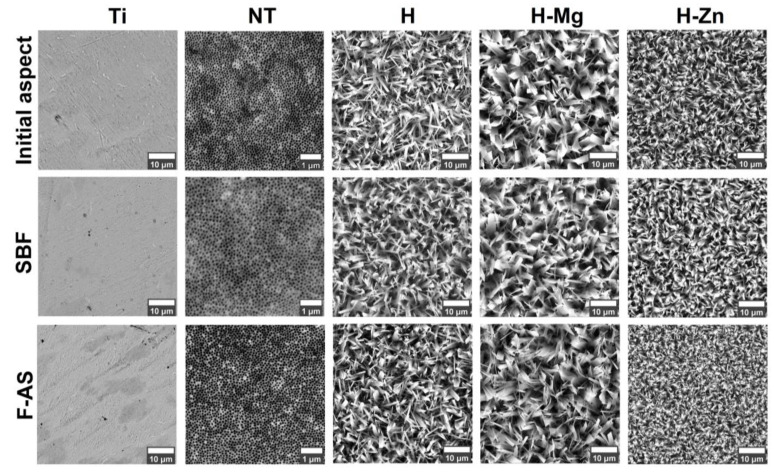
SEM images with the coating’s morphology after the electrochemical tests in SBF and F-AS.

Following the electrochemical tests, the investigated materials were morphologically analyzed with a scanning electron microscope (Figure 5), which revealed that the developed materials do not show significant changes regardless of the testing medium, SBF or F-AS. 

### 3.3. Bioactivity

To assess the bioactivity of the newly developed surfaces on pure Ti, biomineralization and biodegradation tests were performed. Ti samples with polished surfaces and nanostructured surfaces with titanium dioxide nanotubes were considered for biomineralization tests in SBF, but they were not used in the biodegradation tests since they are stable in terms of degradation/corrosion in aggressive environments. Since the kinetics of the titanium degradation/corrosion process are relatively low, no visible changes in terms of mass evolution can be seen over the course of the 21 days.

#### 3.3.1. Biomineralization in SBF

**Figure 6:**
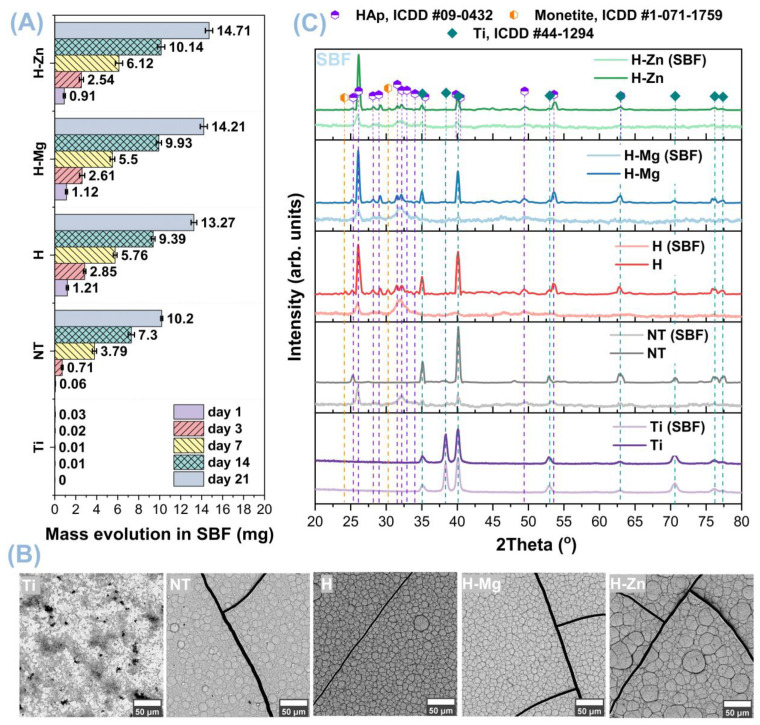
Mass evolution (**A**) of the newly formed apatite on the investigated surfaces, along with SEM images (**B**) and X-ray diffractograms (**C**) of the experimental samples after 21 days of immersion in SBF.

The biomineralization experiments (apatite precipitation) were carried out by immersing the samples in the SBF medium for a duration of up to 21 days. Figure 6 displays the results of the bioactivity tests in terms of the mass evolution of newly precipitated apatite on the surface of the tested materials (Figure 6A) along with the SEM images (Figure 6B) and the diffractograms (Figure 6C) acquired on the investigated surfaces after 21 days of immersion. Based on the results, independent of the immersion period, all materials obtained by biofunctionalization increased the biomineralization ability of the Ti polished surface in simulated body fluid. 

In terms of the mass evolution of the newly formed apatite, it is worth noting that with the exception of Ti, which had the weakest evolution, the uncoated nanostructured surface (NT) had a smaller mass evolution when compared with the one coated with HAp undoped or doped, showing that biofunctionalization with the HAp-based bioactive ceramic layers favored the precipitation of a higher quantity of apatite on the surface. When the masses of undoped HAp-based coatings were compared to those of HAp doped with Mg or Zn, it was revealed that the H-Mg and H-Zn coatings registered values of 2.61 mg and 2.54 mg, respectively, after the first three days of immersion in SBF. However, as the immersion period increased, the coatings doped with Mg or Zn, began to register slightly higher mass values of the newly formed apatite than H, reaching 5.5 mg for H-Mg and 6.12 mg for H-Zn after 7 days, and 9.93 mg and 10.14 mg, respectively, after 14 days of immersion. Thus, after 21 days of immersion, the highest value was 14.71 mg for H-Zn, followed by 14.21 mg for H-Mg and 13.27 mg for H (Figure 6A).

The increased surface free energy of the coatings doped with Mg or Zn (H-Mg, γ_s_^tot^ = 66.9 mN/m; H-Zn, γ_s_^tot^ = 65.8 mN/m) compared with HAp (H, γ_s_^tot^ = 64.7 mN/m) may explain the increase in biomineralization capacity. However, in the case of HAp-based coatings doped with Mg or Zn, this assertion must be coupled with Mg’s increased biodegradability, in comparison with Zn, which leads H-Mg coatings to have a slightly lower biomineralization capacity than H-Zn coatings.

It is commonly assumed that the newly precipitated apatite layer is a distinguishing feature of bioactive materials and that this type of material promotes the formation of a functional and mechanically stable biomaterial–tissue interface more quickly.

The apatite precipitation process on the surface can be interpreted according to the electric charge of the investigated surface. According to the literature, the NT surface [82] has a negative charge at first and begins to attract positive Ca^2+^ ions following immersion in SBF. Once the positive Ca^2+^ ions are present on the entire surface, the resultant material has a positive charge and attracts negative phosphate ions (PO_4_^3−^), favoring the formation of amorphous calcium phosphate, which is metastable and changes over time into crystalline apatite, similar to that found in human hard tissues [83]. 

Although the electrostatic interactions that occur between the functional groups found in the coatings and the calcium and phosphate ions present in the synthetic biological solution commence the process in this case, the entire process is regulated by the features and characteristics of the calcium phosphate coatings [84]. 

SEM images obtained on the polished surfaces (Ti) and the (NT) nanostructured surfaces uncoated and coated with hydroxyapatite undoped and doped with metallic ions (Mg, Zn) after 21 days of immersion in SBF are shown in Figure 6B. It is worth noting that a new layer of apatite was formed in SBF on all surfaces modified by electrochemical techniques, showing a morphology composed of semi-spherical crystals, which is typical for the apatite precipitated from SBF [85].

The layer of the newly precipitated apatite was observed on the entire surface, indicating that the experimental surfaces present superior bioactive properties, as well as a good biomineralization capacity, in SBF in comparison with the pure titanium (Ti) surface.

Regarding the size of the newly formed apatite with a semi-spheroidal shape, it is noted that in the case of H-Zn coatings, their size is larger than those formed on the NT, H, and H-Mg surfaces, which have closer dimensions. Ding et al. [85] showed that Zn-doped HAp-based coatings obtained by electrochemical deposition using the galvanostatic method have a greater biomineralization capacity in SBF than undoped HAp-based coatings. Therefore, it can be stated that the addition of Zn in the HAp structure leads to an improvement in the biomineralization capacity of HAp, with similar results also being obtained by other researchers [86]. 

It may be hypothesized that another factor that impacted the biomineralization ability of HAp-based coatings is the Ca/P ratio, which had lower values. The Ca/P ratios of the coatings (Table 1) are 1.59 for the H coatings, 1.57 for the H-Mg coatings, and 1.56 for the H-Zn coatings before their immersion in SBF. Thus, the coatings with the lowest Ca/P ratio—H-Zn—achieved the highest biomineralization capacity, followed by H-Mg and H. As stated in the literature, Ca-deficient hydroxyapatite (CDHAp) has a greater ability for biomineralization than stoichiometric HAp (Ca/P = 1.67) since its Ca/P ratio is smaller than 1.67 [87,88].

Figure 6C shows the diffractograms obtained for the materials immersed for 21 days in SBF, which were used to identify the type of calcium phosphate formed on the surface. According to the X-ray diffraction results after 21 days of immersion in SBF, the NT surface and the HAp-based coatings undoped and doped show diffraction peaks characteristic for HAp according to the standard ICDD #09-0432, confirming its presence and highlighting that the obtained layers have a bioactive character that can favor osseointegration. Compared with the biofunctionalized surfaces, the Ti samples do not present diffraction peaks specific to calcium phosphates, as confirmed by the gravimetric and SEM analysis.

In the case of the HAp-based coatings, the diffraction peak identified at 2θ = 26° and associated with the (002) diffraction plane presents a diminished intensity, indicating that the precipitated HAp exhibits poor crystallinity compared with the initial one (before immersion in SBF). Moreover, the diffraction peaks found at 2θ of 31.5° and 32.3°, associated with the (211) and (112) planes, form a single diffraction peak after immersion in SBF. At the same time, several diffraction maxima characteristic of monetite, a precursor of hydroxyapatite, can be observed. The diffraction peaks found at 2θ of 24.26° and 30.24° are characteristics of monetite, according to ICDD #1-071-1759. The formation of monetite can be due to the fact that in the case of electrochemical deposition, the formation of calcium phosphates depends on the current density and the pH of the electrolyte. Thus, when a low current density is applied, the pH of the solution near the substrate also decreases, leading to the possibility of precipitation of the monetite phase [89]. During the immersion in SBF, the diffraction maxima of monetite increases in intensity, mostly because of the increment in the pH solution. According to the literature [90], monetite is characterized by a higher resorption speed compared with HAp in SBF, thus releasing Ca^2+^ and PO_4_^3−^ ions faster. In the case of the nanostructured surface, it is noted that the diffraction peak associated with the Ti substrate and titanium dioxide with its two phases, i.e., anatase and rutile, has significantly diminished or even disappeared, while the ones attributed to HAp are clearly visible. The same trend was also observed for the HAp-based coatings, suggesting that the newly formed apatite layer has a notable thickness, which led to smaller intensities of the peaks associated with the Ti substrate.

#### 3.3.2. Biodegradation in PBS 

**Figure 7:**
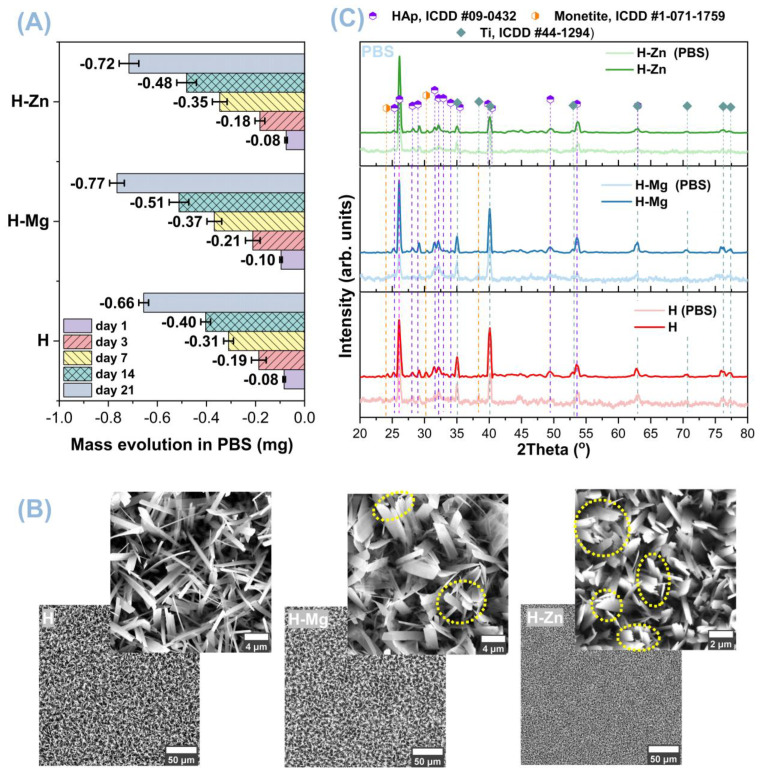
Mass evolution (**A**) of the investigated surfaces, along with SEM images (**B**), in which signs of degradation are identified by yellow circles, and X-ray diffractograms (**C**) of the experimental samples after 21 days of immersion in PBS.

Immersion tests in phosphate buffer solution (PBS) were carried out to estimate the biodegradability of the coatings at different time intervals (1 day, 3 days, 7 days, 14 days, and 21 days). Figure 7A shows the gravimetric results obtained from the biodegradation tests in PBS, based on which it was noted that the addition of the doping elements improved the degradation capacity of HAp. Figure 7B,C show the SEM images and diffractograms obtained for the HAp-based coatings after their immersion for 21 days in PBS. 

The mass evolution results for each period highlighted that after 24 h of immersion in PBS (Figure 7A), the H-Mg coatings have the highest mass loss, of −0.096 mg, followed by the H coatings with −0.083 mg and H-Zn with −0.075 mg. After 3 days of immersion in PBS, the highest mass loss was identified for the H-Mg coatings with −0.211 mg, followed by the H-coatings with −0.187 mg and H-Zn with −0.182 mg. On the seventh day of immersion in PBS, the H-Mg and H-Zn coatings show very close values of −0.368 mg and −0.346 mg, followed by the H ones with −0.310 mg. Starting with on the 14th day, the coatings show the same trend, namely, the H-Mg coatings have the largest mass loss, followed by H-Zn and H. The greatest mass decrement was noted for the H-Mg samples, of −0.765 mg, followed by H-Zn with −0.716 mg, while the H coatings showed a decrease of −0.656 mg, after 21 days of immersion in PBS.

In conclusion, it can be stated that, that the degradation capacity of the HAp-based coatings can be modulated with respect to clinical demands through the addition of Mg or Zn doping elements, which accelerated the degradation processes of the HAp-based layers. 

The coating morphology after immersion in PBS (Figure 7B) confirms the results obtained from the gravimetric analysis. Thus, at higher magnification, it was noted that the ribbon crystals of the H-Mg and H-Zn coatings show a fringed appearance towards the tip, indicating clear signs of degradation.

Figure 7C presents the diffractograms of the coatings before and after immersion in PBS for 21 days, based on which it was observed that the nanostructured surfaces coated with undoped HAp (H) and doped with Mg (H-Mg) or Zn (H-Zn) show a similar appearance to the one before immersion, indicating that the obtained materials are relatively stable and that no other types of phases were obtained in PBS after 21 days of immersion. However, it should be noted that small differences in intensities were observed compared with the initial ones. Thus, it was noted that the HAp-based coatings show a slight decrease in the intensity of the diffraction maximum associated with the (002) plane from 2θ = 26°. This change is more visible in the case of H-Mg coatings, which, according to gravimetric measurements, had the highest mass loss, but also in the case of H-Zn coatings. It was also observed that all coatings show a slight decrease in intensity for the peaks associated with the (211) and (112) diffraction planes at 2θ = 31.77° and 2θ = 32.20°, confirming the degradation process of HAp in PBS.

Since all the coatings investigated by X-ray diffraction after 21 days of immersion in PBS still show diffraction peaks specific to the HAp phase, according to ICDD standard #09-0432, it can be stated that the developed coatings are relatively stable over time. At the same time, it should be emphasized that the addition of biocompatible elements, such as Mg or Zn into the HAp structure, impacts the degradation capacity of HAp, slightly increasing the degradation rate without significantly affecting the morphology and structure of HAp, as demonstrated by the SEM and XRD analyses, respectively.

### 3.4. In Vitro Biological Assay 

#### Cell Seeding and *In Vitro* Biocompatibility Evaluation

**Figure 8:**
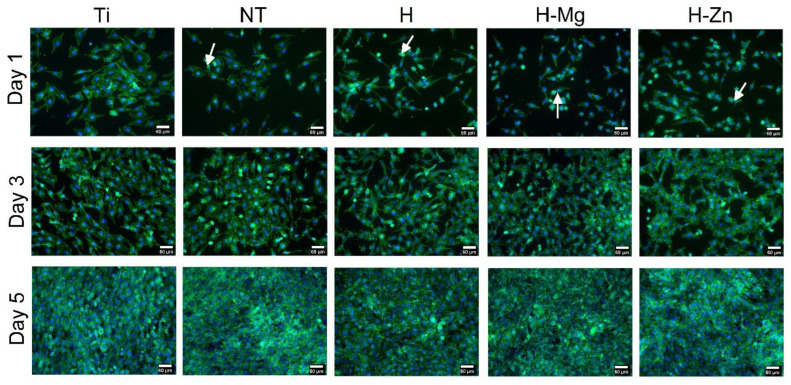
MG62 cells seeded for 1, 3, and 5 days on the investigated materials (white arrows indicate the divided cells).

To monitor the colonization of the experimental samples with osteoblast cells from the MG63 line, fluorescence experiments were performed, allowing for the visualization of the β actin cytoskeleton (green color) and the nucleus (blue color). The polished Ti samples were used as control samples. In Figure 8, it can be observed that 1 day after seeding, the cells adhered to and distributed evenly on all the tested surfaces and started to proliferate (in Figure 8, dividing cells are marked by white arrows), indicating good biocompatibility.

By analyzing the images, it is noted that 1 day and 3 days after seeding with MG63 osteoblast cells, no remarkable differences are observed between the investigated surfaces. Three days after seeding, colonization of the tested samples can be noted in a proportion of approximately 70%, which indicates a good interaction between the MG63 cells and the biomaterials. After 5 days, their surfaces are completely covered by a confluent layer of osteoblasts.

**Figure 9:**
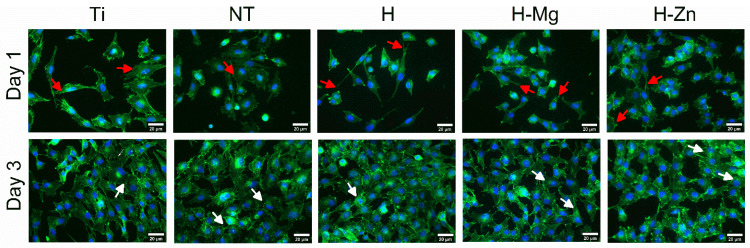
Morphology of the MG63 human osteoblast cell lines analyzed 1 and 3 days after seeding. The cytoskeleton highlights the organization and arrangement of F-actin fibers (white arrows—well-defined actin fibers with dorsal localization, red arrows—prominent actin filaments in the filopodia of cells).

The images obtained at a higher magnification, as shown in Figure 9, indicate that after 24 h of seeding, the cells show a fibroblast-type morphology, specific to osteoblasts, and a normal round-shaped nucleus (Figure 9, white arrows). 

**Figure 10:**
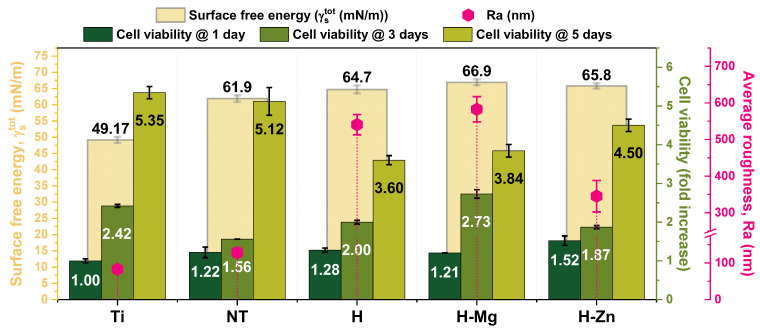
Correlation of cell viability with the surface free energy and surface roughness.

Figure 10 presents the viability tests on MG63 cells seeded on the developed materials at 1, 3, and 5 days, respectively. In comparison with Ti, it is observed that the NT nanostructured surface shows sustained cell adherence and survival, with a 1.22-fold change value after 1 day, 1.56-fold change on the 3rd day, and an increment to a 5.12-fold change after 5 days of seeding, indicating that this substrate can also support cell proliferation. Thus, after 5 days, Ti and the NT surface are comparable in terms of sustaining cell viability and proliferation. In the case of the HAp-based coatings, it is observed that they show different trends. The cells cultured on undoped HAp (H) coatings have a viability of 1.28- and 2.00-folds higher in comparison with the Ti control after 1 and 3 days of seeding, respectively. After 5 days of seeding, the undoped HAp-induced cell viability was 3.60 times higher in the control, which was lower than that of cp-Ti and NT, respectively. This may be due to an induction of osteogenic differentiation by the HAp coating. On the other hand, the addition of the doping elements Mg or Zn into the HAp structure led to higher values than that reached by the undoped HAp, indicating their positive impact on the cell viability and proliferation processes.

By correlating the cell viability results with those obtained for the surface free energy (Figure 2B) and roughness (Table 1), it is observed that although the increment in the surface roughness increases the surface free energy of the investigated materials, not all results confirm this trend (Figure 10). Thus, in the case of the obtained surfaces, it is necessary to take into consideration the morphology of the investigated surfaces since the nanostructured surface (NT) is made of hollow nanotubes with a diameter of 70.08 nm (±11.67 nm) [57,58], which allow for a good anchoring of the cells, while the morphology of the HAp-based coatings is made of ribbon-like crystals. Of course, the chemical composition, especially in the case of the doped HAp-based coatings, cannot be neglected.

Therefore, according to the data presented in Figure 10, it can be noted that the H-Mg coatings show cell viability that is 1.21 times higher than that of Ti after 1 day but that is lower than NT, H, and H-Zn for the same time interval. After 3 days, a sudden increase in the cell viability to a value that was 2.73-fold higher than the control was observed for the H-Mg coatings. After 5 days of seeding, the H-Mg coatings induced a viability that was 3.84-fold higher than the control, which was rather similar to that registered for the undoped HAp but lower than the ones reached for the NT and Ti surface. The cell viability results obtained for the H-Mg coatings are in good agreement with the literature data, which have indicated that Mg^2+^ ions found in Mg-doped HAp favor cell adhesion and proliferation [91,92].

In the case of the Zn-doped HAp-based coatings, it is noted that the cell viability at 1 day and 3 days was 1.52- and 1.87-fold higher than the Ti control. After 5 days of seeding, the H-Zn coatings reached the highest value in terms of cell viability compared with the other HAp coatings, namely, a 4.50-fold change. These results are also in good agreement with other studies [86,93], which have highlighted that in comparison with undoped HAp, the addition of Zn in certain concentrations will enhance cell viability.

The cell viability enhancement after the addition of Zn and Mg is correlated with the degradation process, through which Zn and Mg ions are released from the HAp structure [43,91,94,95]. This is also the case for the proposed experimental coatings, which, after the addition of Zn and Mg, registered higher mass losses in the PBS medium. Another important aspect highlighted by the cell biology assays is that the selected concentrations of Mg and Zn did not have a negative impact on the viability of MG63 osteoblast cells.

### 3.5. Antibacterial Efficiency

**Figure 11:**
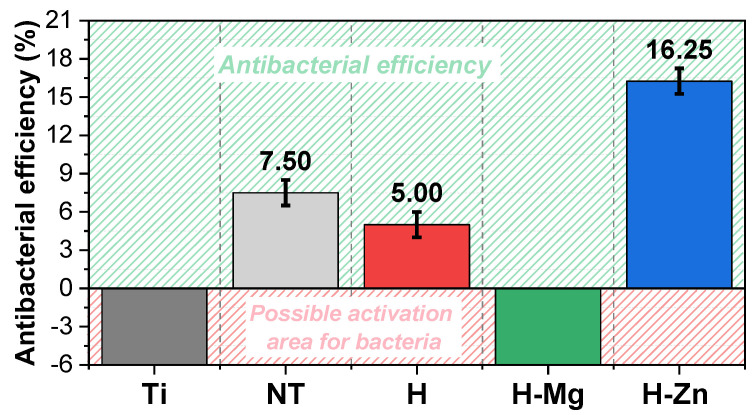
Antibacterial efficiency of the investigated materials for *Escherichia coli*.

Figure 11 presents the results obtained from the antibacterial tests, which highlight that the uncoated nanostructured surface (NT) and the ones coated with undoped (H) and Zn-doped HAp (H-Zn) present some antibacterial characteristics.

In comparison with Ti, which favored the activation of *Escherichia coli* bacteria, the functionalization with titanium dioxide nanotubes leads to a surface with antibacterial characteristics, showing a bacterial efficiency of 7.5%. The results obtained for the NT nanostructured surface are in good agreement with the literature [96,97], which attests that titanium dioxide nanotubes (NTs) show antibacterial efficiency against *Escherichia coli* and also *Staphylococcus aureus*. The study performed by Ercan et al. [98], on the relationship between the size of titanium nanotubes and antibacterial efficacy, showed that TiO_2_ nanotubes with a diameter between 60 and 80 nm significantly decrease the adhesion of bacteria, which is in good agreement with the diameters of the nanotubes obtained for the proposed nanostructured surface (diameter of NT = 70.08 nm (±11.67 nm) [57,58]).

In terms of the HAp-based coatings, the antibacterial tests show that the undoped HAp (H) presents an antibacterial efficiency of 5%. This is most likely due to the nanostructured surface with nanotubes (NTs) since bare HAp does not have an antibacterial effect, which is also the reason why elements with a bactericidal character, such as Zn, Cu, Ag, and Fe, are added.

In contrast, the negative values of the antibacterial efficiency indicate a possible activation of *Escherichia coli* bacteria. Thus, it can be noted that the addition of Mg at the proposed concentration into the HAp structure does not induce an antibacterial effect. Similar results for Mg-doped HAp coatings have also been reported in other studies [99,100]. 

However, it cannot be neglected that there are also studies that attest that at high concentrations, Mg^2+^ ions show antibacterial properties [101]. Mg concentration plays an important role in the antibacterial efficiency of Mg-containing materials, as most bacterial cells contain a Mg concentration ranging between 15 and 30 mM [45]. Therefore, when Mg ions are used as a single antibacterial agent, their concentration must be higher than that present in bacteria cells, although it is estimated that even a moderate concentration, together with a basic/alkaline pH of the environment (minimum pH of 8), can determine a good antibacterial effect [102]. 

Knowing that Mg can induce an antibacterial effect when a minimum concentration of 30 mM is used and comparing it with the one used to obtain the H-Mg coatings of 1 mM in the electrolyte, it is obvious that the latter concentration is 30 times lower. Thus, it can be assumed that the unfavorable antibacterial result registered for the H-Mg coatings is a direct cause of the Mg concentration used.

Regarding the H-Zn coatings, it is noted that the addition of Zn has an enhanced antibacterial effect on *Escherichia coli* bacteria, registering an antibacterial efficiency of 16.25%, which is also the highest in the tests performed. The obtained results are in good agreement with other studies [99,103]. 

Although the exact mechanism through which Zn has an antibacterial effect is not fully understood, a possible explanation could be that Zn^2+^ ions penetrate the bacterial cell membrane, producing reactive oxygen species (ROS) that disrupt the multiplication activity of deoxyribonucleic acid (DNA). Zn^2+^ is also able to bind to the sulfhydryl groups (-SH) of metabolic enzymes, disrupting the respiratory activity of bacteria and leading to their lysis [104]. The interaction between Zn^2+^ ions and bacterial cells is attributed to the surface electric charge, which, in the case of Zn, is positive, while bacterial cells have a negative charge. The surface charge difference enhances the attraction between the two by creating electrostatic forces that lead to a strong ionic bond between Zn and the bacterial surface [105].

According to a study carried out by Sergi et al. [103], it was highlighted that HAp-based coatings with Zn show a better antibacterial efficiency on *Staphylococcus aureus* bacteria, which are Gram-positive, rather than on Gram-negative ones such as *Escherichia coli*, since Gram-negative bacteria present an additional outer membrane (outside the peptidoglycan layer) that behaves as a physical barrier that can interfere with the released Zn^2+^ ions [106]. Based on this, it can be assumed that the proposed coatings will also offer antibacterial efficiency against *Staphylococcus aureus*. 

In conclusion, it can be said that the nanostructured surface uncoated and coated with undoped and Zn-doped HAp provides good antibacterial efficiency, while the addition of Mg leads to the possible activation of bacteria, showing poor antibacterial efficiency. It is also noted that the H-Zn coatings showed an antibacterial efficiency against *Escherichia coli* that is twice that of the uncoated nanostructured surface (NT) and three times that of the undoped HAp-based coatings (H).

## 4. Conclusions

In this paper, Mg- and Zn-doped HAp-based coatings were successfully deposited on titanium dioxide nanotubes by the pulsed galvanostatic method to enhance the biological features of the Ti substrate. In comparison with the bare Ti substrate, all developed surfaces enhanced the surface free energy and the biomineralization ability, with the highest values being registered for the doped HAp-based coatings. The electrochemical tests carried out in SBF and F-AS pointed out that the best behavior was reached by the undoped HAp-based coatings, followed by the ones doped with Zn and the ones doped with Mg, indirectly indicating that through the addition of the doping elements, the solubility of the HAp-based coatings can be controlled. The bioactivity tests achieved in SBF media showed that the biomineralization ability of the bare Ti surface can be enhanced through the presence of TiO_2_ NTs uncoated and coated with HAp-based coatings, with the highest quantity of the newly formed apatite being registered for the HAp coatings doped with Zn, closely followed by the ones doped with Mg. 

The cell culture tests carried out with osteoblast cells from the MG63 line indicated that after 5 days of seeding, the highest cell viability among the HAp-based coatings was reached for the ones containing Zn-doped HAp coatings, which also showed the highest antibacterial efficiency, followed by the ones doped with Mg. Nonetheless, these tests also showed that the TiO_2_ NT surfaces uncoated and coated with undoped HAp also present some antibacterial efficiency.

## Figures and Tables

**Table 1:** The main physico-chemical properties of the hydroxyapatite-based coatings undoped and doped with Mg or Zn [57,58].

Samples	H	H-Mg1	H-Mg2	H-Zn1	H-Zn2
Ref.	[57,58]	[58]	[58]	[57]	[57]
Ca/P ratio	1.59	1.56	1.55	1.54	1.52
(Ca + M)/P, ratio M = Mg or Zn	1.59	1.57	1.56	1.56	1.55
Mg content in hard bone tissue (0.32–0.78 wt.%)	−	0.46 at.% (0.31 wt.%)	0.54 at.% (0.37 wt.%)	−	−
Zn content for antibacterial efficiency (~1.2 wt.%)	−	−	−	0.78 at.% (1.39 wt.%)	1.30 at.% (2.30 wt.%)
Crystallite dimension, L_002_ (nm)	19.96 nm	26.11 nm	26.41 nm	25.60 nm	25.47 nm
Crystallinity, (%)	20.90%	46.94%	48.56%	44.25%	43.60%
Average roughness, Ra (nm)	540 nm	583 nm	494 nm	319 nm	240 nm
Coating thickness [μm]	9.75 (±0.91) μm	11.3 (±0.6) μm	13.7 (±1.1) μm	10.5 (±0.85) μm	5.5 (±0.7) μm
Adhesion ASTM D-3359-17 [59] 5B: 0%; 4B: ≤5% 3B: 5%	Delaminated area (%)				
	0.31%	0.44%	5−15%	0.55%	5−15%
	4B (good)	4B (good)	3B (average)	4B (good)	3B (average)

**Table 2:** Electrolyte chemical composition and sample codification.

Substrate		Pure Ti biofunctionalized with titanium dioxide nanotubes (NTs TiO_2_)		
Coating material		Undoped HAp	HAp doped with Mg	HAp doped with Zn
Sample codification		H	H-Mg	H-Zn
Electrolyte (mM)	Ca(NO_3_)_2_·4H_2_O	10.000	9.000	9.985
	NH_4_H_2_PO_4_	6.000		
	Mg(NO_3_)_2_·6 H_2_O	0	1.000	0
	Zn(NO_3_)_2_·6H_2_O	0	0	0.015
pH		Adjusted to 5 by the dropwise addition of 1M NaOH		

**Table 3:** Characteristics of the different employed liquids.

	Deionized Water	Ethylene Glycol	Di-Iodomethane
$\gamma_{s}^{d}$	21.8 mN/m	29.0 mN/m	50.8 mN/m
$\gamma_{s}^{p}$	51.0 mN/m	19.0 mN/m	-
SFE	72.8 mN/m	48.0 mN/m	50.8 mN/m

**Table 4:** The electrolytes’ chemical composition used to evaluate the electrochemical behavior.

SBF		F-AS	
Reagent	Amount	Reagent	Amount
NaCl	8.035 gL^−1^	NaCl	0.4 gL^−1^
NaHCO_3_	0.350 gL^−1^	KCl	0.9 gL^−1^
KCl	0.225 gL^−1^	urea	1 gL^−1^
K_2_HPO_4_·3H_2_O	0.231 gL^−1^	NaH_2_PO_4_	0.69 gL^−1^
MgCl_2_·6H_2_O	0.311 gL^−1^	CaCl_2_·2H_2_O	0.795 gL^−1^
1 M-HCl	39 mL	Na_2_S·9H_2_O	0.005 gL^−1^
CaCl_2_	0.292 gL^−1^		
Na_2_SO_4_	0.072 gL^−1^		
(CH_2_OH)_3_CNH_2_	6.118 gL^−1^		
pH 7.4		pH 5.2	

**Table 5:** Electrochemical parameters for the investigated materials in SBF and SA.

Media	Sample	E_corr_ (mV)	i_corr_ (nA/cm^2^)	β_c_ (mV)	β_a_ (mV)	Rp (kΩ × cm^2^)
SBF	Ti	−177.19	226.45	180.93	155.05	160.31
	NT	−132.26	74.77	100.21	990.71	529.19
	H	−87.62	52.92	123.97	249.32	680.23
	H-Mg	−213.30	65.51	106.03	474.09	575.15
	H-Zn	−115.03	57.72	100.41	1067	691.35
F-AS	Ti	−68.55	83.66	498.25	842.32	1626.97
	NT	14.13	25.41	125.87	876.76	1883.56
	H	−33.08	10.74	86.28	751.89	3133.22
	H-Mg	14.77	18.74	81.53	613.58	1669.38
	H-Zn	−21.67	12.54	122.44	109.98	2008.91

## Data Availability

The data are contained within this article.
